# Combustion control of DME HCCI using charge dilution and spark assistance

**DOI:** 10.1177/09544070221103361

**Published:** 2022-06-07

**Authors:** Xiao Yu, Simon LeBlanc, Navjot Sandhu, Jimi Tjong, Ming Zheng

**Affiliations:** Department of Mechanical, Automotive, and Materials Engineering, University of Windsor, Windsor, ON, Canada

**Keywords:** DME, renewable fuel, HCCI, SACI, lean burn, charge dilution

## Abstract

To realize the potential of DME for clean combustion, fueling control is essential. In this research, the challenges, advantages, and applicability of high-pressure direct injection and low-pressure port injection are reviewed and evaluated, especially in relevance to HCCI combustion. In this study, emphasis is given to the applicable ranges of low-pressure fuel delivery in relevance to load, air-fuel ratio, and inert gas dilution, for realizing HCCI combustion. The strategy of high-pressure direct injection is advantageous for combustion phasing control, but the fuel handling is challenging because of the high vapor pressure of DME fuel. The strategy of port fuel injection is prone to early combustion and, consequently, tends to produce excessive pressure rise rates in the combustion chamber. This challenge is escalated at higher engine loads, making homogenous charge compression ignition difficult to achieve. In this paper, the load extension of DME-fueled HCCI combustion was explored. First, the impact of dilution on the combustion characteristics of DME HCCI was studied under lean and CO_2_ diluted conditions. Under the present empirical setups, results show that the lean-burn strategy has limited capability of combustion phasing control, especially when the engine load is above 5 bar IMEP. The CO_2_ dilution strategy can significantly retard the combustion phasing until the fulfillment of combustion becomes unstable. It was found that spark assistance is advantageous for combustion control. With an effective application of excess air, intake CO_2_ dilution and spark assistance, an engine load of 8 bar IMEP was reached with appropriate combustion phasing, with ultra-low NO_x_ emissions.

## Introduction

The transportation sector is one of the largest consumers of petroleum-based energy sources in the world.^1^ The high energy density and convenient handling of liquid hydrocarbon fuels make them suitable as energy sources for automotive applications. Petroleum-based fuels powering the internal combustion engines could be progressively blended with or replaced by renewable fuels with lower carbon footprints to minimize net CO_2_ emissions from the transportation sector.^2,3^

Dimethyl ether (DME) has shown the potential to reduce net greenhouse gas (GHG) emissions while maintaining similar engine efficiency as compared to diesel.^4–6^ DME can be produced via methanol dehydration or syngas synthesis, a process that use renewable feedstocks such as biomass.^7,8^ In this way, renewable DME can largely negate engine-out CO_2_ emissions from its combustion as it is then considered a part of the biomass life cycle.

DME has been used for decades for many applications such as domestic heating, power generation, a propellant for aerosol cans, and most recently, transportation.^9^ As a fuel, DME was first applied as a supporting fuel to improve the cold-start operation of methanol engines owing to its low boiling point.^10,11^ Since then, most empirical investigations have used DME as a neat or blended fuel with diesel for direct injection (DI) compression ignition (CI) engines.^12,13^

Generally, the CI engines operate with diesel-fueled, high-pressure direct injection systems. This conventional application ensues heterogeneous combustion, which is efficient but produces excessive nitrogen oxides (NO_x_). As a result, various combustion strategies have been developed to limit NO_x_ and avoid the characteristic soot-NO_x_ trade-off, such as EGR enabled low temperature combustion (LTC) and multi-pulse fuel scheduling. However, combustion strategies that lower combustion temperature drastically are prone to reduced combustion efficiency because of the unburned HC and CO emissions in the exhaust.^14^

The application of alternative fuels shows the potential to overcome this challenge. Table 1 presents important fuel properties comparing diesel and gasoline fuel with renewable alternatives, including DME and ethanol fuel.

**Table 1.** Properties for selected automotive fuels.^15,16^

Property		Diesel	DME	Ethanol	Gasoline
Chemical formula	-	C_n_H_1.87n_	C_2_H_6_O	C_2_H_6_O	C_n_H_1.77n_
Cetane number	-	40–50	>55	5–20	8
Stoichiometric air/fuel ratio	-	14.6	9	9	14.2
Energy density (LHV)	MJ/kg	42.5	28.4	26.9	44
Oxygen content	mass %	0	34.8	34.8	0
Liquid kinematic viscosity	cSt	3	<0.1	1.2–1.5	0.5–0.6
Liquid density @ 15°C	kg/m^3^	831	667 (5 bar)	785–810	750–765
Auto-ignition temperature	°C	210	180	363	246–280
Vapor pressure	kPa	≪10	530	17	53–60
Normal boiling temperature	°C	177–370	−24.8	78	27–225

The soot-free nature of DME combustion is contributed by the high oxygen concentration, the lack of carbon-to-carbon bonding, and a low boiling point.^17,18^ The high volatility of DME fuel enhances fuel mixing quality, leading to higher mixture homogeneity before the combustion process.^19^ Despite the advantages of the combustion and emission characteristics of DME compared with diesel, the fuel handling of DME fuel for high pressure direct injection application faces significant challenges because of the physical properties of DME, such as high vapor pressure, high compressibility, poor lubricity, and its corrosive nature to elastomers.^20^ Specially designed high pressure direct injection DME fueling systems have been tested for on-road application, but these systems are relatively complicated as compared to conventional diesel fuel injection systems.^21,22^ Compared with high pressure direct injection strategies, port fuel injection (PFI) strategy is easier to realize because of the lower injection pressure (up to 20 bar). However, the low autoignition temperature of DME results in early combustion, engine knocking and low engine efficiencies.

The primary challenge for HCCI combustion is the lack of combustion control because the ignition of the air-fuel mixture is dominated by the chemical kinetics of the charge mixture.^23^ The heat-producing low-temperature reactions are prominent in low octane fuels, leading to earlier autoignition compared to the high octane fuels.^24^ This drawback escalated under increased engine loads wherein higher in-cylinder temperatures further advance the onset of autoignition. As such, the engine load of HCCI research fueled by high reactivity fuels such as DME is typically below 5 bar IMEP.^25–27^ However, studies on HCCI combustion using less chemical reactive fuels have reported much higher engine loads, especially under intake boosted conditions. For example, Dec and Yang^28^ reported gasoline-fueled HCCI tests near 16 bar IMEP under 14:1 CR and 3.25 bar intake boost. Additionally, Christensen et al.^29^ reported natural gas-fueled HCCI tests of 15.6 bar IMEP under 17:1 CR and 2.5 bar intake boost.

Several methodologies have been investigated to minimize the compression temperatures in order to retard the autoignition timing, such as reducing engine compression ratio (CR).^30^ Jung and Iida^31^ studied the control of DME HCCI combustion at 4 bar IMEP using EGR in a single-cylinder engine with an engine CR of 10.76:1. It was observed that the combustion phasing window was knocking-limited in the early phasing, while stability-limited at the late phasing of combustion. Under a lower compression ratio, both the in-cylinder pressure and temperature during compression stroke were reduced, which increased the indicated thermal efficiency because of the more favorable combustion phasing (CA50).^32^ Prior research conducted at the authors’ laboratory investigated DME HCCI.^33,34^ The engine load was increased from 1.1 bar IMEP at 17.8:1 CR to 2.5 bar IMEP under 13.1:1 CR with much lower peak pressure and delayed combustion phasing (2–3CA).^33^

Another approach to control the autoignition timing involves intake charge dilution using excess-air (lean operation) or EGR.^26,35,36^ Nakano et al.^37^ presented an in-depth investigation into the effects of EGR on HCCI using engine simulation and detailed chemical reaction models. The results showed that the use of EGR could delay the ignition timing so long as the EGR did not increase the charge temperature, affirming the importance of intake charge temperature cooling when EGR was used.^37^ In this work, the intake charge was dosed with CO_2_ to dilute the intake charge and behave to control the combustion phasing.

In this work, the challenges, advantages and applicability of high-pressure direct injection and low pressure port injection are reviewed and evaluated, especially in relevance to HCCI combustion. A 9.2:1 CR was used to ensure sufficient delay in the autoignition timing of DME HCCI, while both excess air and intake dosed CO_2_ were used to control the phasing of DME combustion. The emphasis is given to the applicable ranges of low-pressure fuel delivery in relevance to load, air-fuel ratio and inert gas dilution to realize HCCI and spark assisted compression ignition (SACI) combustion. A balance of strategies is employed to extend the load of DME HCCI beyond those of currently published works.

## Experimental setups

**Figure 1.**
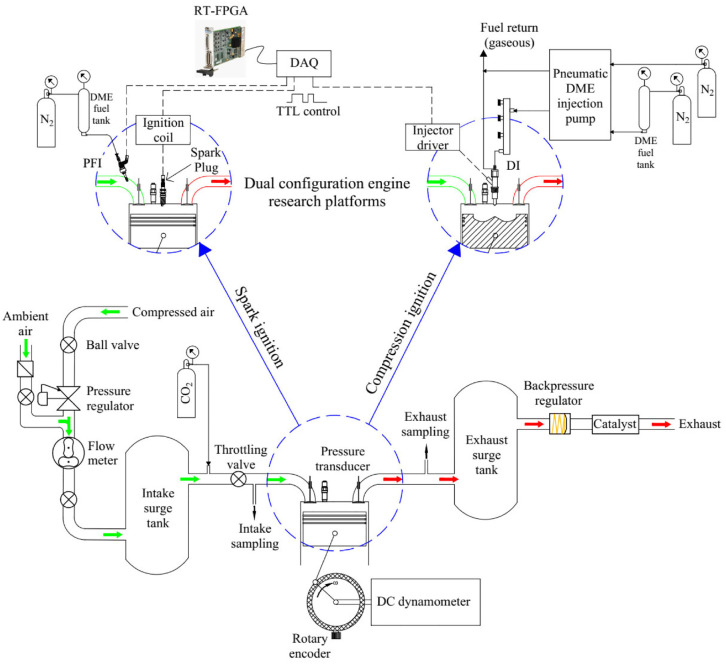
Single-cylinder engine platform for spark ignition and compression ignition research.

The tests were conducted using two single-cylinder research engine platforms, as shown in Figure 1. A high CR engine was used for high pressure direct injection (DI) studies while a low CR engine was used for port fueled DME HCCI combustion studies. Dry compressed air was used to emulate the engine boost with an electro-pneumatic pressure regulator to control the intake manifold pressure. A roots-type flow meter was installed between the regulator and surge tank to measure the volumetric flow rate of fresh air supply. The equivalent MAF was calculated via the measured volumetric flow rate and the local air density. Both the engine coolant and engine oil temperature were controlled using external FEV coolant/lubricant conditioning units. The research engine specifications are presented in Table 2.

**Table 2.** Test engine specifications.

Fueling system	Direct injection	Port fuel injection
Engine type	Single-cylinder, 4-stroke	Single-cylinder, 4-stroke
Engine speed	1200 rpm	1300 rpm
Fuel pressure	600 bar (up to 1400 bar)	20 bar
Displacement	744 cm^3^	857 cm^3^
Bore	95 mm	102 mm
Stroke	105 mm	105 mm
Compression ratio	16.5:1	9.2:1
Piston bowl	Deep omega	Shallow bowl

The spark and fueling commands were controlled using National Instruments (NI) real-time (RT) controllers embedded with field-programmable gate array (FPGA) devices. The commands were generated by the FPGA system and sent to the ignition coil and injector power drivers. The fuel injection and spark timing were crank-angle resolved at a resolution of 0.1°CA, while the coil charging duration and the fuel injection duration were time-resolved at a time resolution of 25 ns. The in-cylinder pressure was measured with a flush-mounted piezoelectric pressure transducer. The charge signal was amplified and recorded inside the RT controller. About 200 consecutive engine cycles were recorded under each operating condition and the average pressure trace was used for analysis.

### Direct injection

The physical properties of DME, such as high vapor pressure, low viscosity (i.e. susceptible to vapor bubble formation or cavitation) and being corrosive to elastomers, render conventional rotary-type injection pumps unfit for DME fuel without significant modifications. Alternatively, plunger-type pumps can withstand these fuel properties and generate high fuel injection pressures. In this work, a double-air drive single-acting pneumatic pump system was used to establish high fuel injection pressure. Specifications of the system are summarized in Table 3.

**Table 3.** DME injection pump specification.

Type	Pneumatic plunger pump
Model	LSF100-2
Configuration	Double-air drive piston, single-acting
Pressure ratio	226:1
Fuel pressure	Up to 1600 bar
Seal material	Polytetrafluoroethylene (PTFE)
Stroke volume	9 mL

The pump was self-lubricated and tailored with polytetrafluoroethylene (PTFE) seals. Nitrogen was supplied as the driving gas for the pneumatic pump. The driving pressure was regulated to control the fuel injection pressure. The DME fuel tank was pressurized up to 8 bar using Nitrogen to ensure liquid fuel was supplied to the high-pressure pneumatic pump.

In operation, the driving piston inside the pneumatic cylinder applied force through a rod to the fuel reservoir by the plunger, which increased the fuel pressure. As the fuel was consumed through injections, the piston continued its path progressively further downward until the fuel reservoir was empty, at which point air was released from the pneumatic cylinder, pushing the driving piston back to the top, simultaneously filling the fuel reservoir. This event is referred to as *pump recycling*. This phenomenon limits the duration of stable high fuel injection pressure as the pump recycling induces pressure fluctuations to the common rail.

**Figure 2.**
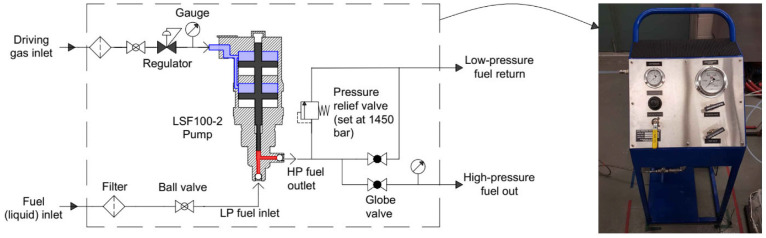
Fueling system for high pressure DME.

The fuel injection system was fitted with filters, valves, and regulators, as shown in Figure 2. In this setup, the driving air was directed to a regulator wherein the setpoint of the output fuel pressure could be controlled. The high-pressure fuel outlet line was split into a pressure relief valve, a low-pressure vent valve, and a high-pressure fuel outlet valve. During operation, the vent valve was fully closed.

### Port injection

The investigation on the combustion control of DME was performed with a port fuel injection system to guarantee sufficient air-fuel mixing. The fuel injection pressure was set to 20 bar to maintain DME in a liquid phase. A GDI injector was fitted into the intake manifold near the intake valve for fuel delivery. For all tests, the injection timing was fixed at 10°CA ATDC during the intake stroke. A 3785 cm^3^ stainless steel tank was used to store DME. Compressed N_2_ gas was used to generate the 20 bar DME for liquid fuel supply. The solubility of N_2_ into liquid DME was negligible toward the combustion behavior.

An iridium spark plug was centrally mounted on the flat cylinder head as the ignition source. The focus of this study was to investigate the characteristics of DME combustion under a range of excess-air and intake CO_2_ dilution levels. Two approaches were taken to conduct this study. To study the impact of excess-air ratio on HCCI combustion characteristics, the fuel flow rate was fixed while the mass air flow (MAF) rate was increased under each excess-air ratio. Alternatively, to study the impact of CO_2_ dilution, mass flow rates of both fuel and intake air were fixed when CO_2_ gas was dosed into the intake manifold. The quantity of CO_2_ dosed into the intake manifold was monitored by the intake analyzer feed and controlled by a pressure regulator and flow control valves.

### Emission analysis

The intake and exhaust gases were analyzed by a pair of California Analytical Instruments (CAI) 600 series emission analyzer benches. Before entering the analyzers, the sampled exhaust gas passed through a conditioning unit to eliminate water vapor and particulate matter entering the analyzer systems. The emission analyzers included a flame ionization detector (FID) for total hydrocarbon (C_1_) measurement, a nondispersive infrared (NDIR) sensor for CO and CO_2_ analysis, a paramagnetic oxygen sensor for intake and exhaust O_2_ measurement, and a heated chemiluminescence detector (CLD) for NO_x_ measurements. The relevant specifications of each sampled gas are tabulated in Table 4.

**Table 4.** Emission analyzer specifications.

	Species	Range	Accuracy
Non-dispersiveinfrared	CO	0%–1%	0.001%
	CO_2_	0%–20%	0.020%
Paramagneticdetection	O_2_	0%–25%	0.025%
Chemiluminescence	NO & NO_2_	0%–3000 ppm	0.01 ppm
Flame ionizationdetection	HC	0%–20,000 ppm	0.01 ppm

The combustion efficiency was calculated from the supplied fuel energy, and exhaust energy from CO and HC emissions, as shown in equation (1).

(1) $$\begin{array}{c} \eta_{\mathrm{comb}}=\left(1-\;\frac{{\overset{\cdot }{m}}_{\mathrm{HC}}\left(\mathrm{LH}V_{\mathrm{HC}}\right)+{\overset{\cdot }{m}}_{\mathrm{CO}}\left(\mathrm{LH}V_{\mathrm{CO}}\right)}{{\overset{\cdot }{m}}_{f}\cdot \mathrm{LH}V_{f}}\;\right)\\ \;x100\% \end{array}$$

where, ${\overset{\cdot }{m}}_{f}$ is the fuel flow rate (g/s), $\mathrm{LH}V_{f}$ is the energy density of the fuel (28.4 MJ/kg), ${\overset{\cdot }{m}}_{\mathrm{HC}}$ and ${\overset{\cdot }{m}}_{\mathrm{CO}}$ are the measured mass flow rates of HC and CO in the exhaust (g/s), while $\mathrm{LH}V_{\mathrm{HC}}$ and $\mathrm{LH}V_{\mathrm{CO}}$ are the energy densities of HC (assumed to be the supplied fuel, $\mathrm{LH}V_{f}$ ) and CO (10.2 MJ/kg), respectively.

The apparent heat release rate (AHRR) was calculated from in-cylinder pressure based on the first law of thermodynamic fundamentals, as shown in equation (2).

(2) $$\frac{dQ_{\mathrm{app}}}{d\theta }=\;\frac{1}{\gamma -1}\left[\gamma \cdot p\cdot \frac{\mathrm{dV}}{d\theta }\;+V\cdot \frac{\mathrm{dp}}{d\theta }\right]$$

where, $\frac{dQ_{\mathrm{app}}}{d\theta }$ is the AHRR (J/°CA), γ is the specific heat ratio of air (assumed constant at 1.37), p is the in-cylinder pressure, and V is the cylinder volume.

The AHHR was used to estimate the combustion phasing through normalized cumulative heat release. The start of combustion (SOC) and end of combustion (EOC) was approximated to 5% (CA05) and 95% (CA95) cumulative heat release, respectively. The combustion duration (CD) was the duration between the SOC and EOC. The ignition delay (ID) was defined as the period from spark timing to CA05. The combustion phasing was expressed as the midpoint of heat release, 50% (CA50).

## Results and discussions

### Direct injection

#### Spray study

The spray behavior of DME fuel was recorded with a high-speed camera in a 3 L constant volume combustion vessel fitted with heating elements for background temperature control. N_2_ was used as the background media for all test cases. The DME fuel injection system (Figure 2), was connected to a piezoelectric 8-hole injector with a nozzle diameter of 180 μm. In this application, the pneumatic pump was ideal to deliver a stable fuel pressure.

**Figure 3.**
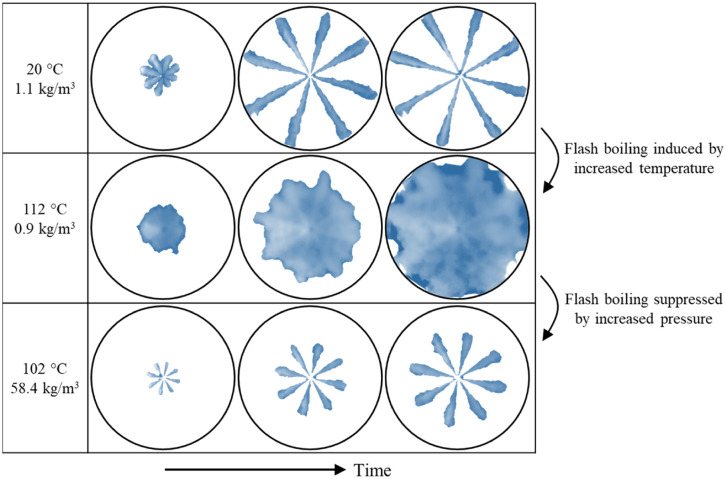
DME fuel spray into inert background gas (N_2_) at 600 bar fuel pressure and 1000 μs injection duration.

The high vapor pressure and low boiling temperature of DME is helpful for in-cylinder mixing. Especially when DME fuel is sprayed into a heated background at atmosphere pressure, flash boiling can be observed after leaving the nozzle hole, as shown in Figure 3.

**Figure 4.**
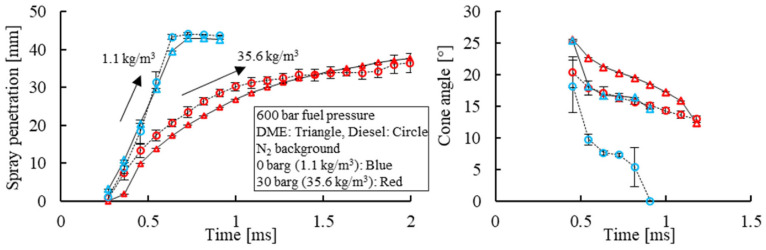
Spray penetration and cone angle of diesel and DME fuel.

The physical properties of DME are distinct from that of diesel. As such, dissimilar spray characteristics become apparent, as shown in Figure 4. The markers represent an average of six repeated test points, with error bars representing the respective standard deviation. The spray cone angle of DME is 15° under atmospheric background density, compared with that of diesel near 5°. Specifically, DME has a lower density and higher volatility which increases the spray cone angle.^38^ Under ambient background pressure, the self-boiling of DME further enhances the vaporization as compared with diesel which remains as liquid. Under increased background density, the background gas interaction with the fuel spray increased and the air entrainment was enhanced, leading to a wider diesel spray cone angle.

#### Engine application

The engine studies with high pressure direct injection strategies were tested on the single-cylinder research engine with a compression ratio of 16.5:1. Contrary to the spray studies which involved infrequent injection events, that is, once every few minutes, engine studies required frequent injections, resulting in *pump recycling*. The cyclic nature of the plunger pump system depended on the injection pressure, engine load, and engine speed. For example, under 1200 rpm engine speed and 600 bar fuel injection pressure, pump recycling occurred every 8 s (80 engine cycles) for 450 μs injection duration and 4 s (40 engine cycles) for 1000 μs injection duration. Consequently, fuel injection pressure fluctuations of 40 bar led to 3%–5% COV of IMEP over a 200 continuous cycle data recording.

**Figure 5.**
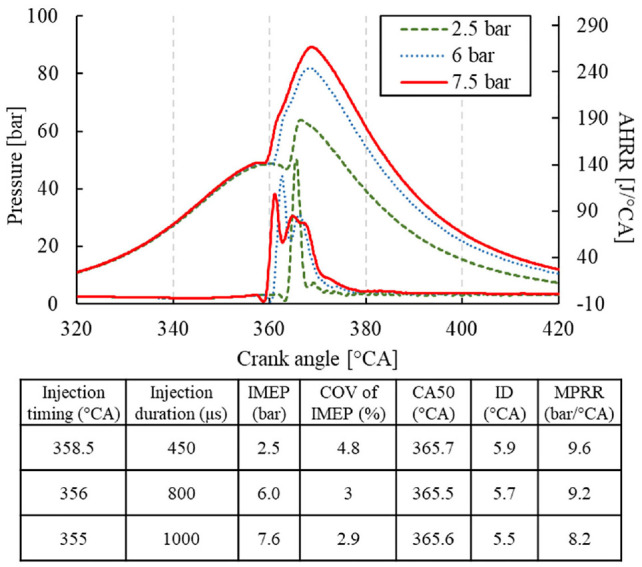
Single-shot direct injection of DME up to 1000 μs injection duration.

The single-shot experiments were first performed to compare the combustion behavior under various engine loads. The combustion phasing was fixed near 366 ã A by adjusting the injection timing, as shown in Figure 5. The high reactivity of DME fuel resulted in a short ignition delay. As the fuel was sprayed into the combustion chamber and ignition ensued, the premixed portion of DME combusted, followed by continued fuel delivery and thus a second combustion period, clearly observable at the longest injection duration cases (7.5 bar IMEP).

**Figure 6.**
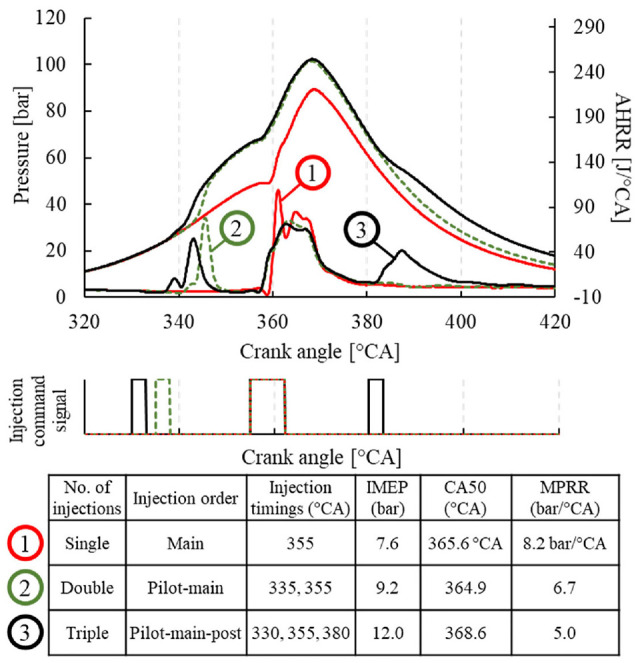
Multi-shot direct injection of DME up to 12.1 bar IMEP.

The same*-*cycle multi-shot injection strategy has become a common practice in real applications. Early injection facilitates a reduced combustion noise through a smoother pressure rise rate at the start of combustion. Opposingly, a late injection can reduce engine-out emissions and increase engine load without increasing peak in-cylinder pressure. Such fuel scheduling was tested with neat DME, as shown in Figure 6. Engine loads up to 12.1 bar IMEP was achieved through a triple shot injection strategy showing low in-cylinder pressure rise rate and low smoke emissions ( <0.01 g/kWh). To achieve higher engine loads, injector modifications would be necessary to avoid extended injection durations, such as increased nozzle hole diameter.

Because of the high chemical reactivity of DME fuel, the high-pressure direct injection strategy is ideal for combustion control, especially under high load operating conditions. However, modifications in high pressure pumps and fuel injectors are necessary to adapt to the high pressure up to 40 bar in the fuel return loops to maintain the DME in the liquid phase during the engine operation. Additionally, the nozzle diameter of the injector needs to be specially designed to deliver a sufficient amount of fuel for full load engine operation.

### Port injection

The principal focus of this paper was to study the controllability of homogeneous combustion of DME-air mixture. The high chemical reactivity of DME triggers auto-ignition with an advanced combustion phasing, which demands intake dilution techniques to control the combustion process. Three sets of experiments were performed, focusing on the impact of engine load, intake dilution, and spark assistance on the DME combustion process. With an effective application of excess air and CO_2_ dilution, along with spark assistance, an engine load of 8 bar IMEP was achieved with preferable combustion phasing and low NO_x_ emissions.

#### Intake dilution impact

**Figure 7.**
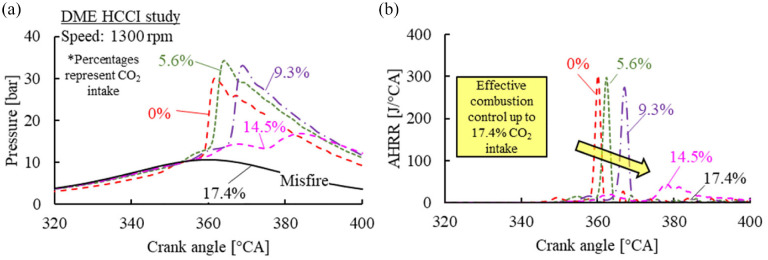
The (a) in-cylinder pressure and (b) apparent heat release rate traces of DME HCCI with CO_2_ intake dilution.

The impact of intake CO_2_ concentration on in-cylinder pressure and AHRR traces of DME HCCI was investigated first, as shown in Figure 7. The intake CO_2_ concentration was increased until the DME mixture ceased autoignition, while the mass flow rates of fuel and air were fixed. The impact of intake CO_2_ concentration was mainly attributed to the decreased oxygen concentration and lowered compression temperature, both contributing to delayed low-temperature reactions.^35^ The autoignition of the DME-air mixture was suppressed when the intake CO_2_ concentration reached 17.4%.

**Figure 8.**
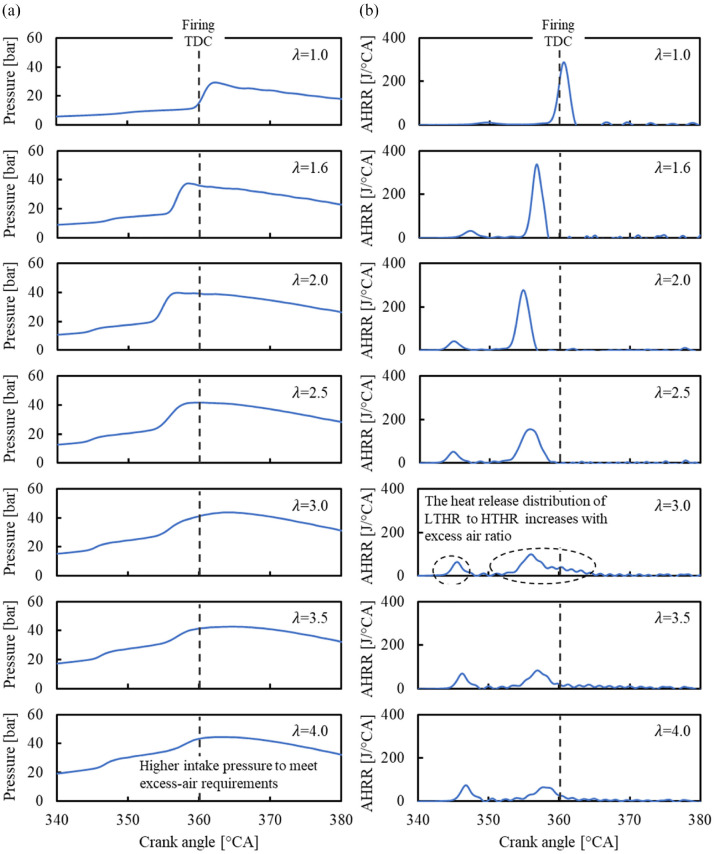
The (a) in-cylinder pressure and (b) apparent heat release traces for DME HCCI with excess-air (λ) intake dilution.

**Figure 9.**
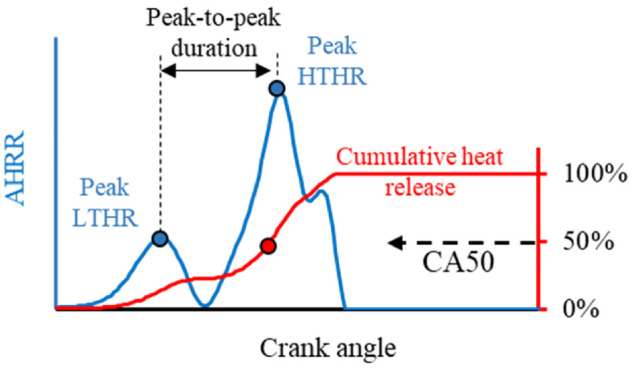
Definition of combustion analysis parameters derived from apparent heat release analysis.

The impact of excess air dilution on the in-cylinder pressure and AHRR is shown in Figure 8. Under stoichiometric conditions, DME showed little low temperature reactions followed by a rapid release of heat from high-temperature reactions. The first and second stages were termed low-temperature heat release (LTHR) and high-temperature heat release (HTHR) respectively. To quantify the behavior of both heat release stages, the apex of each stage was recorded. The timing and delay between both stages were emphasized for comparison, as demonstrated in Figure 9.

The two-stage heat release behavior became more obvious with the increase in the excess-air ratio, as marked in Figure 8(b). The phasing of LTHR was advanced with the increase of excess-air ratio until 2.0, then stayed fixed near 344°CA as the excess-air ratio increased. The phasing of HTHR followed a similar trend as LTHR because the increased in-cylinder temperature and radical concentration generated during the LTHR likely promoted the HTHR, thereby shaping the HTHR timing.

The energy distribution released by fuel became increasingly LTHR-dominant with the increase in excess-air ratio. The increased intake manifold pressure likely assists in the earlier combustion phasing. This phenomenon was best observed from an excess-air ratio of 1 to 2. Beyond an excess-air ratio of 2, the intensity of high-temperature reactions further reduced while the phasing of LTHR remained constant. The HTHR was significantly weakened at an excess-air ratio of 4 as the combustion process became unstable because of the low in-cylinder temperature and mixture dilution.

**Figure 10.**
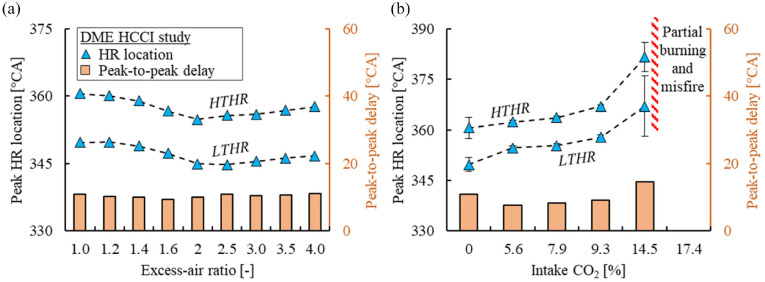
The impact of (a) excess-air ratio and (b) intake CO_2_ concentration on the two-stage (LTHR and HTHR) heat release behavior.

Contrary to the impact of excess air dilution, the heat release of both LTHR and HTHR retarded unidirectionally with the increase of CO_2_ intake concentration, as shown in Figure 10. The phasing of the HTHR was governed by the LTHR phasing. The CO_2_ dilution was effective in retarding the combustion phasing of DME HCCI. Additionally, the heat released during LTHR decreased with the increase in CO_2_ concentration. The delay between the LTHR and HTHR peaks extended with intake CO_2_ concentration and appeared more sensitive than excess air dilution.

**Figure 11.**
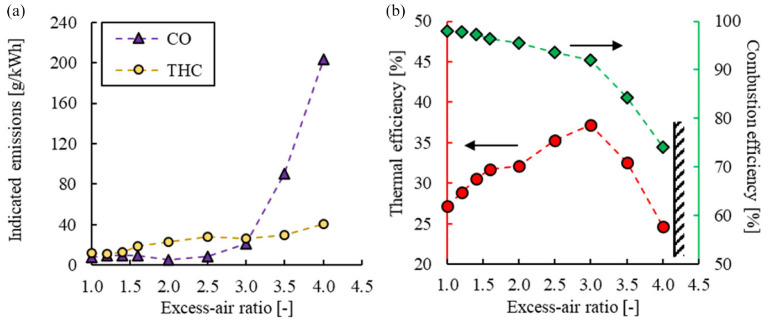
The (a) indicated CO and THC emissions and (b) cycle efficiency (indicated thermal and combustion) for excess-air intake dilution.

The combustion efficiency drops with the increase of excess-air ratio, as shown in Figure 11. A gradual decrease in combustion efficiency with increasing thermal efficiency was observed from stoichiometric to an excess-air ratio of 3. The indicated efficiency may benefit from multiple aspects related to lean burn operation, including lower combustion temperature, higher intake pressure, and higher specific heat ratio of the working fluid. Beyond an excess-air ratio of 3, the combustion efficiency decreased significantly, clear by the sharp rise in CO emissions in Figure 11(a). Consequently, the thermal efficiency declined proportionally to the combustion efficiency.

**Figure 12.**
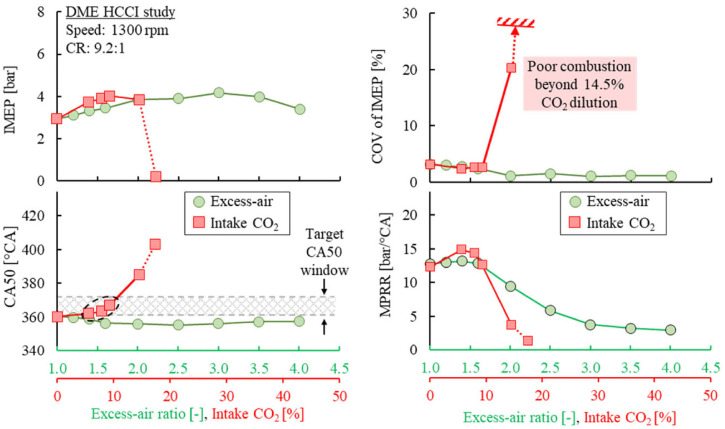
The combustion performance and behavior for DME HCCI with excess air and intake CO_2_ dilution.

Among both dilution methods, CO_2_ dilution primarily impacts the combustion process by two key factors: (1) the reduced intake O_2_ concentration and (2) the reduced in-cylinder compression temperature. On the other hand, excess-air dilution can be extended significantly without notably deteriorating the combustion efficiency. Figure 12 shows the impact of both dilution methods on the engine load (IMEP), engine stability (COV of IMEP), combustion phasing (CA50), and maximum pressure rise rate (MPRR). The performance of lean DME combustion showed little suppression as the engine load and stability improved throughout. Extending the excess-air radio advanced the combustion phasing only slightly, albeit remaining too early to achieve preferable thermal efficiency, for example 365–375 °CA.^39,40^

**Figure 13.**
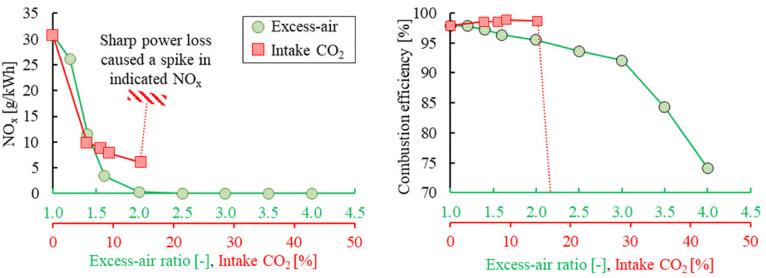
The indicated NO_x_ emission and combustion efficiency with excess air and intake CO_2_ dilution.

The intake CO_2_ dilution showed strong combustion phasing control capability. Under 10% intake CO_2_ concentration, the combustion phasing retarded by 25°CA resulting in an increased engine load. Beyond 14.5% CO_2_, the autoignition of the DME charge mixture was significantly suppressed and combustion efficiency sharply dropped, as shown in Figure 13. Lean DME HCCI could achieve low NO_x_ emissions near 0.01 g/kWh at 92% combustion efficiency. A further improvement over the lean-burn combustion regime requires further delayed combustion timing. Likewise, CO_2_ dilution also showed an impact on engine-out NO_x_ emissions, but the decrement was limited because of the dilution limit.

**Figure 14.**
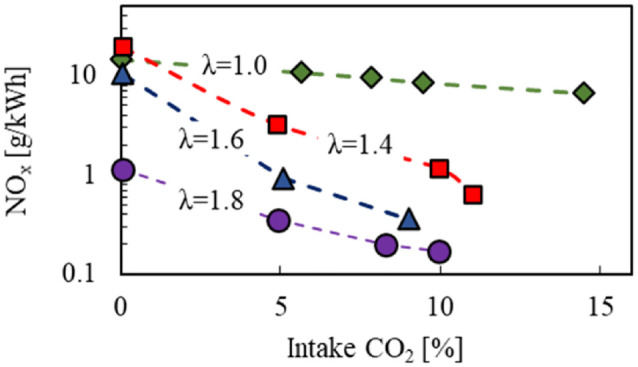
Simultaneous application of excess-air and CO_2_ dilution to reduce NO_x_ emissions.

Figure 14 shows the impact of intake CO_2_ concentration on engine-out NO_x_ emissions under various excess-air ratios. Note that the specific NO_x_ emissions are presented on a logarithm scale for clear description. The NO_x_ emissions under lean-burn (excess-air ratio >2) reduced up to 90% compared with a stoichiometric mixture using CO_2_ dilution. The drop in NO_x_ emissions can be correlated to the lowered peak bulk gas temperature under lean-burn combustion,^41^ demonstrating the potential of dual-dilution modulation for optimized combustion of DME with low combustion temperatures.

#### Engine load study

**Figure 15.**
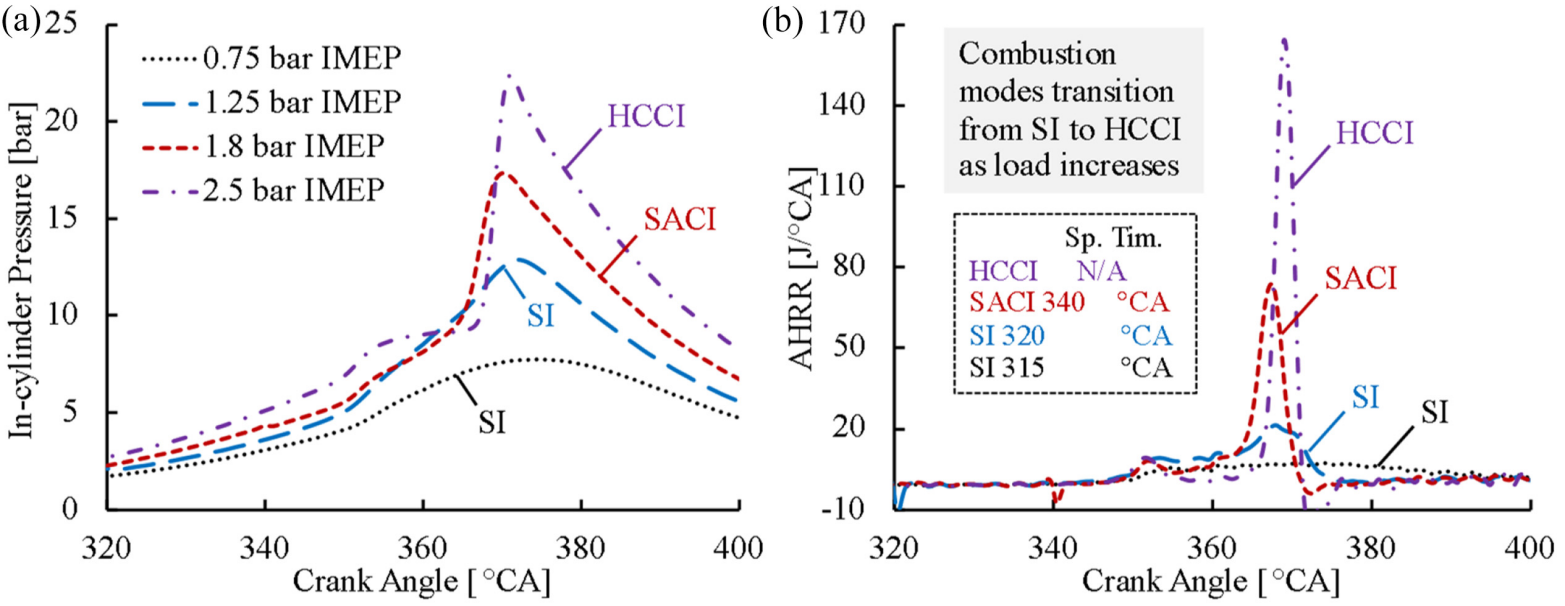
The (a) in-cylinder pressure and (b) apparent heat release rate traces of stoichiometric DME at low load.

The impact of engine load on DME combustion was first investigated under low load stoichiometric conditions. Figure 15 shows the in-cylinder pressure and AHRR traces of DME under various load conditions ranging from 0.75 to 2.5 bar IMEP. Initially, below 1.25 bar IMEP, the DME mixture could not reach autoignition because of the low in-cylinder compression temperature. In such cases, a spark event was necessary to initiate the combustion process. At 1.8 bar IMEP engine load, the autoignition of DME was evident prior to the spark event. The combustion phasing could be controlled with spark timing by initiating flame propagation before the onset of autoignition – a spark-assisted compression ignition (SACI) mode of combustion – to realize suitable combustion timing.

**Figure 16.**
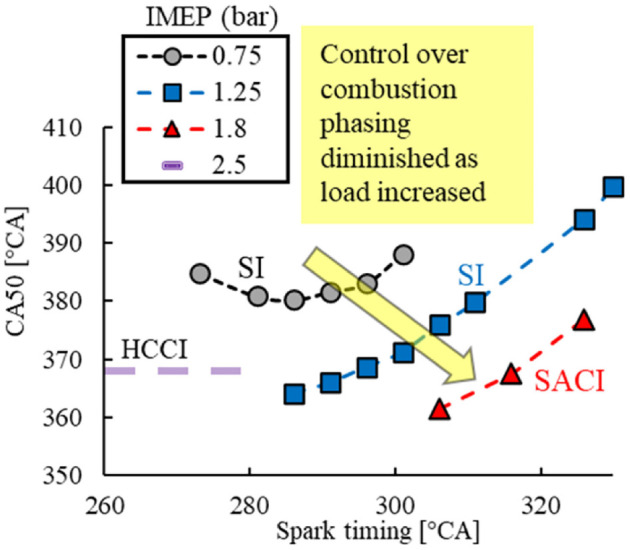
Combustion phasing control of stoichiometric DME combustion at low load.

The impact of spark timing on combustion phasing was investigated under each engine load, as shown in Figure 16. The sparking event had limited capability of combustion phasing control under an engine load of 0.75 bar IMEP, mainly a result of the limited effective spark timing window. With the increase in engine load, the spark event was more effective in controlling the combustion timing over a much wider range of spark timings. On the other hand, the in-cylinder conditions under higher engine loads promoted autoignition, which rendered the spark assistance redundant as observed at 2.5 bar IMEP. This trend was followed through into higher engine loads, which required intake dilution strategies to delay the autoignition point for spark assistance to be applicable.

**Figure 17.**
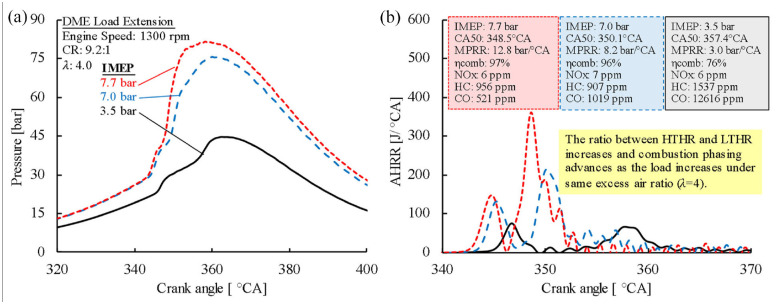
The (a) in-cylinder pressure and (b) apparent heat release rate traces of DME at increasing engine loads. The excess-air ratio was fixed to 4.0.

The engine load of HCCI combustion is commonly limited by early combustion phasing. Under a similar excess-air ratio, higher engine loads promoted LTHR and thus advancing the combustion phasing, as shown in Figure 17. As a direct consequence, a significant increase of in-cylinder pressure and pressure rise rate were apparent. This combustion behavior is a leading factor to why current DME HCCI experimental studies have only reported up to 5 bar IMEP.^26,27^

#### HCCI combustion control

Early autoignition is the principal limitation of high load HCCI combustion. This limitation is especially challenging with highly reactive fuels such as DME. Therefore, to enable DME HCCI combustion with preferable combustion phasing, the onset of low-temperature reactions needs to be controlled.^23^

In the previous discussions, it was observed that excess-air dilution was effective in extending engine load and suppressing NO_x_ emissions, yet had a limited impact on combustion phasing. On the other hand, CO_2_ dilution was effective in delaying combustion and lessening pressure rise rates, however, the dilution amount was limited to combustion instability. The simultaneous application of both dilution techniques showed significant suppression in combustion temperatures over that of their independent applications (recall Figure 14). The chemical reactivity of the in-cylinder mixture needs to be properly modulated, with a spark event serving as a necessary strategy to initiate the combustion process. This technique has shown positive assistance in combustion control in many HCCI control publications.^42,43^

**Figure 18.**
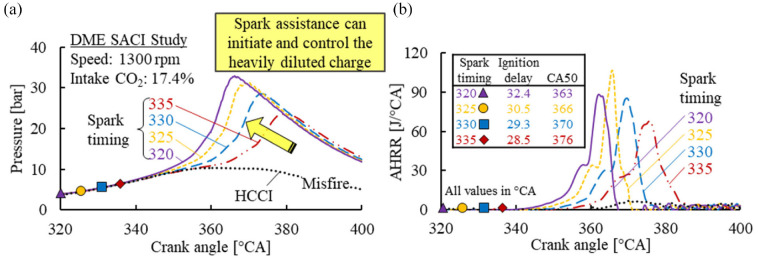
The (a) in-cylinder pressure and (b) apparent heat release traces for DME SACI. The intake CO_2_ was fixed at 17.4%.

As previously shown in Figure 4, CO_2_ dilution was very effective in suppressing the onset of DME HCCI combustion. Therefore, under 17.4% intake CO_2_ dilution concentration, a spark event was necessary to initiate the combustion process, as shown in Figure 18. Through spark assistance, the otherwise misfiring cycle could be successfully initiated with a robust control over the combustion phasing using the spark timing.

**Figure 19.**
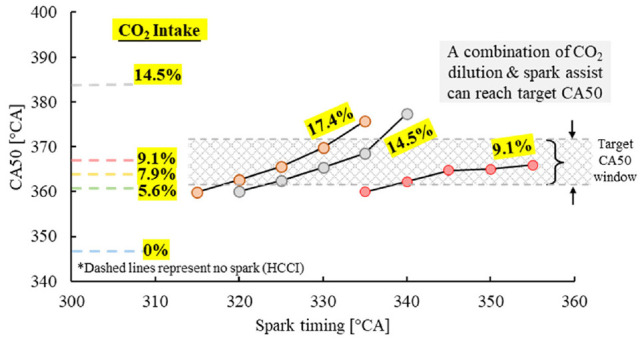
Controlling the combustion phasing of DME SACI with CO_2_ dilution and spark timing.

It should be noted that the spark event could only advance the combustion phasing earlier than the autoignition point of the mixture, as shown in Figure 19. The dashed lines nearest to the Y-axis represent the combustion phasing of the HCCI cases under various intake CO_2_ dilution levels, which represent the latest combustion phasing achievable under those conditions. Higher CO_2_ dilution levels showed increased effectiveness in combustion control, as observed by the steeper slopes from 9.1% to 17.4% CO_2_.

#### Load extension

**Figure 20.**
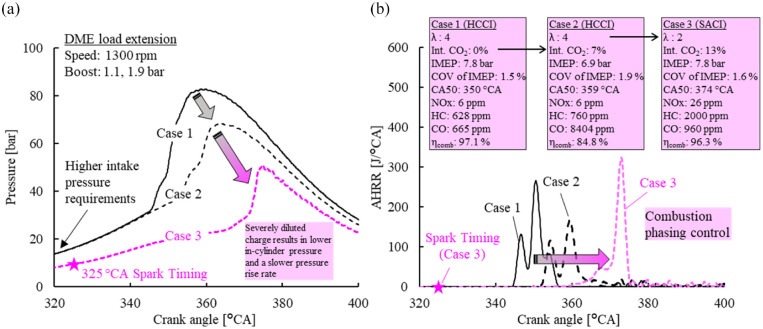
Extending engine load of DME HCCI combustion with a combination of excess air and CO_2_ dilution, along with spark assistance. (a) In-cylinder pressure curve, (b) Heat release rate curve.

The application of all three strategies was demonstrated to best control the combustion of DME HCCI under extended engine loads. Figure 20 shows three cases of DME homogeneous combustion under engine loads close to 8 bar IMEP. The baseline condition was a lean-burn operation of an excess-air ratio of 4.0 at 7.8 bar IMEP (Case 1). Very early combustion phasing was observed with ultra-low NO_x_ emissions and moderate HC/CO emissions. To retard the combustion phasing, the intake was diluted with 7% intake CO_2_ (Case 2). The combustion phasing was consequently retarded by 9 °CA toward TDC. A drop in engine load of 0.9 bar IMEP was observed along with nearly 25% lower in-cylinder peak pressure. Moreover, a significant increase in exhaust CO emissions was evident, leading to a sharp decrease in combustion efficiency.

Lastly, a spark event was introduced to allow higher CO_2_ dilution and retarded combustion phasing under a similar engine load (Case 3). The combination of three strategies was coordinated for optimal combustion phasing at 7.8 bar IMEP: intake CO_2_ concentration of 13%, excess-air ratio of 2.0 and spark timing of 325 °CA. This combination provided a similar engine load as Case 1 while having improved combustion phasing, combustion efficiency, and thermal efficiency, which lowered boost requirements. Engine stability showed negligible changes among the selected cases. Combustion timing after TDC likely experienced higher bulk gas combustion temperatures and led to the marginal increase in NOx emissions.^44^ Future research will push the engine loads further and enhance combustion efficiency under a moderately higher engine compression ratio and dilution ratio.

## Conclusion

The characteristics and controllability of DME in HCCI mode were investigated under various conditions, including engine load, intake dilution, and spark assistance. The appropriate application of the fuel-air control strategies demonstrated the potential of extending engine load limitations of premixed combustion using fuels with high chemical reactivity, such as DME. The primary conclusions of this work are summarized as follows:

1. For DME HCCI combustion, intake CO_2_ dilution showed a strong ability to delay the autoignition timing and retard combustion phasing. An increase in the excess-air ratio, on the other hand, showed a limited impact on the combustion phasing and promoted the low-temperature heat release.
2. Port-fueled DME at low engine load operation ( <1.8 bar IMEP) required a spark event for successful combustion. As the engine load increased, the increased cylinder pressure and temperatures led to autoignition of DME charge mixture for HCCI combustion. In this region, spark ignition could advance the combustion timing. This technique was rendered ineffective at higher engine loads because of the early autoignition of DME charge.
3. A combination of CO_2_ dilution and lean-burn strategy could significantly reduce NO_x_ emissions of stoichiometric DME HCCI combustion. Spark assistance became necessary to initiate the combustion process when the chemical reactivity of the mixture was decreased by significant CO_2_ dilution.
4. The simultaneous application of charge dilution and spark assistance proved capable of achieving near 8 bar IMEP at appropriate combustion phasing. The SACI combustion mode allowed preferable combustion phasing with a decrease in the intake boost demand, while maintaining similar engine performance.
